# Supplementary material for: An image segmentation technique with statistical strategies for pesticide efficacy assessment
Source: PLoS One. 2021 Mar 15;16(3):e0248592. doi: 10.1371/journal.pone.0248592 (PMC7959351; doi:10.1371/journal.pone.0248592)
Supplement: S1 Appendix — (DOCX) [file pone.0248592.s002.docx]

**S1 Appendix**

Let µ be the expected proportion of green area (which should be estimated given observed proportions in the data). To model µ as a function of D, P_0_, and P_1_ using the beta regression, we let h(µ) = ln[µ / (1 – µ)] and assume h(µ) = α_0_ + α_1_ P_0_ + α_2_ P_1_ + β_0_ D + β_1_ (D × P_0_) + β_2_ (D × P_1_).

For the control (i.e., P_0_ = 0; P_1_ = 0), at D = *d* days after treatment, the beta regression can be expressed as h(µ) = α_0_ + β_0_ *d*, and the inverse function is

$\mu_{C}(d)=\frac{e^{\alpha_{0}+\beta_{0}d}}{1+e^{\alpha_{0}+\beta_{0}d}}$

We define the adjusted proportion on day *d* as

$\theta_{C}(d)=\frac{\mu_{C}\left( d \right)-\mu_{C}(0)}{1-\mu_{C}(0)}$

so that *θ_C_* (*d*) → 0 as d → 0 and *θ_C_* (*d*) → 1 as d → ∞ assuming the expected proportion of green area increases with respect to days after treatment. By algebra, the adjusted proportion can be expressed as

$\theta_{C}\left( d \right)=\frac{\frac{e^{\alpha_{0}+\beta_{0}d}}{1+e^{\alpha_{0}+\beta_{0}d}}-\frac{e^{\alpha_{0}}}{1+e^{\alpha_{0}}}}{1-\frac{e^{\alpha_{0}}}{1+e^{\alpha_{0}}}}$

$$=\frac{\left( e^{\alpha_{0}+\beta_{0}d} \right)\left( 1+e^{\alpha_{0}} \right)}{1+e^{\alpha_{0}+\beta_{0}d}}-e^{\alpha_{0}}$$

$=\frac{\left( e^{\alpha_{0}+\beta_{0}d} \right)\left( 1+e^{\alpha_{0}} \right)-\left( 1+e^{\alpha_{0}+\beta_{0}d} \right)e^{\alpha_{0}}}{1+e^{\alpha_{0}+\beta_{0}d}}$

$=\frac{e^{\alpha_{0}+\beta_{0}d}-e^{\alpha_{0}}}{1+e^{\alpha_{0}+\beta_{0}d}}$

which is Eq 1.

For the nongray zone of HP (i.e., P_0_ = 1; P_1_ = 0), at *d* days after treatment, the beta regression can be expressed as h(µ) = (α_0_ + α_1_) + (β_0 +_ β_1_) *d*, so the inverse function is

$\mu_{0}(d)=\frac{e^{\alpha_{0}+\alpha_{1}+\left( \beta_{0}+\beta_{1} \right)d}}{1+e^{\alpha_{0}+\alpha_{1}+\left( \beta_{0}+\beta_{1} \right)d}}$

which has the same functional form except α_0_ + α_1_ instead of α_0_ and β_0_ + β_1_ instead of β_0_. Using the same algebraic procedure to derive Eq 1, the adjusted proportion can be expressed as

$\theta_{0}\left( d \right)=\frac{e^{\alpha_{0}+\alpha_{1}+\left( \beta_{0}+\beta_{1} \right)d}-e^{\alpha_{0}+\alpha_{1}}}{1+e^{\alpha_{0}+\alpha_{1}+\left( \beta_{0}+\beta_{1} \right)d}}$

which is Eq 2.

Finally, for the gray zone of HP (i.e., P_0_ = 0; P_1_ = 1), at *d* days after treatment, the beta regression can be expressed as h(µ) = (α_0_ + α_2_) + (β_0 +_ β_2_) *d*, so the inverse function is

$\mu_{1}(d)=\frac{e^{\alpha_{0}+\alpha_{2}+\left( \beta_{0}+\beta_{2} \right)d}}{1+e^{\alpha_{0}+\alpha_{2}+\left( \beta_{0}+\beta_{2} \right)d}}$.

Using the same algebraic procedure,

$\theta_{1}\left( d \right)=\frac{e^{\alpha_{0}+\alpha_{2}+\left( \beta_{0}+\beta_{2} \right)d}-e^{\alpha_{0}+\alpha_{2}}}{1+e^{\alpha_{0}+\alpha_{2}+\left( \beta_{0}+\beta_{2} \right)d}}$

which is Eq 3.
